# Tactile priming modulates the activation of the fronto-parietal circuit during tactile angle match and non-match processing: an fMRI study

**DOI:** 10.3389/fnhum.2014.00926

**Published:** 2014-12-15

**Authors:** Jiajia Yang, Yinghua Yu, Akinori Kunita, Qiang Huang, Jinglong Wu, Nobukatsu Sawamoto, Hidenao Fukuyama

**Affiliations:** ^1^Biomedical Engineering Laboratory, Graduate School of Natural Science and Technology, Okayama UniversityOkayama, Japan; ^2^Intelligent Robotics Institute, School of Mechatronical Engineering, Beijing Institute of TechnologyBeijing, China; ^3^Key Laboratory of Biomimetic Robots and Systems, Ministry of EducationChina; ^4^Human Brain Research Center (HBRC), Kyoto University Graduate School of MedicineKyoto, Japan

**Keywords:** delayed match to sample task, tactile spatial working memory, priming effect, fMRI

## Abstract

The repetition of a stimulus task reduces the neural activity within certain cortical regions responsible for working memory (WM) processing. Although previous evidence has shown that repeated vibrotactile stimuli reduce the activation in the ventrolateral prefrontal cortex, whether the repeated tactile spatial stimuli triggered the priming effect correlated with the same cortical region remains unclear. Therefore, we used event-related functional magnetic resonance imaging (fMRI) and a delayed match-to-sample task to investigate the contributions of the priming effect to tactile spatial WM processing. Fourteen healthy volunteers were asked to encode three tactile angle stimuli during the encoding phase and one tactile angle stimulus during the recognition phase. Then, they answered whether the last angle stimulus was presented during the encoding phase. As expected, both the Match and Non-Match tasks activated a similar cerebral network. The critical new finding was decreased brain activity in the left inferior frontal gyrus (IFG), the right posterior parietal cortex (PPC) and bilateral medial frontal gyri (mFG) for the match task compared to the Non-Match task. Therefore, we suggest that the tactile priming engaged repetition suppression mechanisms during tactile angle matching, and this process decreased the activation of the fronto-parietal circuit, including IFG, mFG and PPC.

## Introduction

Working memory (WM) is the cognitive operation that underlies our ability to temporarily maintain information in the mind to guide future behavior. Recently, many neuroimaging studies (for review, see Zimmer, 2008) have focused on the neural activity related to information coding and the maintenance of visual WM. Although vision is the most dominant sensory modality, humans also rely on tactile and kinesthetic information to explore object features, such as texture or shape. Some researchers have used neuroimaging approaches to assess the neural mechanisms of tactile (Kostopoulos et al., 2007; Kaas et al., 2013) and haptic (Kaas et al., 2007) WM. The results of those studies indicated that, except in specific areas, such as the primary somatosensory cortex (SI) and the primary visual cortex (V1), the WM processing of different sensory modalities activated very similar brain networks. The common neuronal substrates of WM for different modalities are primarily the bilateral frontal and prefrontal cortex (PFC), the medial frontal gyrus (mFG) and posterior parietal cortex (PPC), which together are referred to as the fronto-parietal circuit. One previous study (Ricciardi et al., 2006) has indicated that both the visual and haptic systems utilize similar processing streams subserving perception and WM compared to the visual system. However, the neural processes underlying tactile or haptic WM are not fully understood.

The delayed match to sample task is a widely used test of WM in humans (Yoon et al., 2006; Fiehler et al., 2008; Zanto et al., 2011). In general, the task requires a subject to first encode a sample stimulus. After a short delay, a stimulus is presented during the recognition phase, and the subject is asked to make a forced-choice response to determine whether these two stimuli are matched or non-matched. Usually, researchers change the number of stimuli during the encoding phase (Blokland et al., 2008; Leung and Alain, 2011) or the length of the delay period between the encoding and recognition phase (Kaas et al., 2007) to test the brain activation related to the maintenance of sensory information or memory load. These two factors change the brain activity seen during WM processing in patterns that are widely recognized in the field.

The repetition of a stimulus during the task often decreases neural activity within certain cortical regions. This phenomenon is known as repetition suppression or the priming effect (for review, see Schacter et al., 2004). The brain will process a stimulus more quickly and/or correctly if the stimulus has been experienced before. During the past two decades, many neuroscientists have focused on the neural processes underlying the priming effect, establishing that priming is generally associated with decreased cortical activity (for review, see Schacter et al., 2007). Although this evidence was mostly obtained from visual and auditory priming studies, a recent study (Burton et al., 2012) that used repeated vibrotactile stimuli confirmed the priming effect in tactile modality. Specifically, Burton et al. (2012) found activity reductions in the ventrolateral prefrontal cortex but not in the SI of sighted subjects after repeated trials.

Both vibrotactile and spatial stimuli activate similar brain areas, however, the temporally specific tactile information of vibrotactile stimuli (such as vibrotactile sequence or frequency) is likely to be coded separately from the tactile spatial information (Lederman and Klatzky, 1997; Bodegård et al., 2000, 2001). In particular, evidence from a recent fMRI study (Li Hegner et al., 2010) indicated that the activation of the right PPC was stronger during tactile pattern discrimination than during a tactile frequency task. Therefore, we hypothesized that the priming of tactile spatial stimuli would lead to reduced activation in regions of the common fronto-parietal circuit, such as IFG and mFG. In contrast with repeated vibrotactile stimuli (Burton et al., 2012), the brain activation may also decrease in the right PPC, which is functionally specific for tactile spatial processing.

In the current study, a modified delayed match-to-sample task was used to investigate how the priming effect influences neuronal substrates of tactile spatial WM processing and to test our hypothesis. We used a restricted working definition of tactile spatial stimuli that can be applied to any object with angles, which has been used in our previous studies (Wu et al., 2010; Yang et al., 2012). During functional magnetic resonance imaging (fMRI), the subjects were asked whether a tactile angle stimulus presented during the recognition phase appeared during the encoding phase. To visualize the tactile priming effect, we directly compared the brain activation during a non-match task to a match task.

## Materials and methods

### Subjects

Fourteen right-handed healthy male volunteers aged 23–31 years (mean age 24.6 ± 0.71 years) participated in the fMRI study. Before the start of the experiment, all subjects participated in a training session outside of the MR scanner in which they were instructed to perform all the procedures in the protocol. All subjects gave their informed written consent, and this study was approved by the Ethics Committee of Human and Animal Experiments, Kyoto University and Okayama University, Japan.

### Tactile stimuli

**Figure 1:**
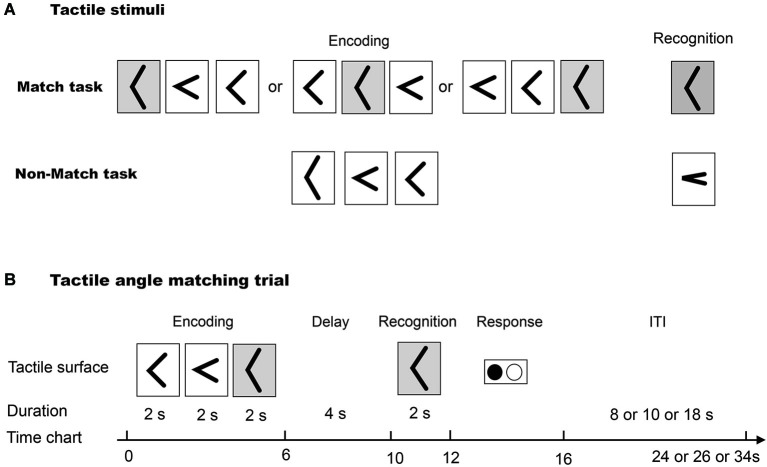
**(A)** The tactile angle stimuli for Match and Non-Match tasks. **(B)** The diagram illustrates one trial paradigm for the tactile angle matching task.

Five raised angles (30, 60, 90, 120 and 150°, as in our previous study (Wu et al., 2010)) were used in this study (Figure 1A). These raised angles consisted of custom-built plastic shapes that were raised 0.5 mm over a 40.0 mm square base, and the arms were 8.0 mm long and 1.5 mm wide, as described in our previous study (Wu et al., 2010).

### Procedure

We used an event-related fMRI paradigm to assess task-related neuronal activity during the tactile angle matching task. The subjects lay supine in the MRI tunnel with earplugs and were instructed to relax. The subjects’ right arms were extended to the device and were comfortably supported by cushions. The subjects placed their right index fingertips lightly on the surface, and the other fingers rested on a plastic frame. The left index and middle fingers were placed on the response box.

As illustrated in Figure 1B, first, three angle stimuli were moved under the subjects’ index fingers. The subjects were asked to perceive and remember the size of each angle stimulus during a 6 s encoding phase. Then, after a 4 s delay phase, one angle stimulus was presented to the index finger. The subjects were asked to perceive it during a 2 s cognition phase. Lastly, the subjects were asked to identify whether the last angle stimulus had been presented during the encoding phase by using the response key during a 4 s response phase. The total duration of one tactile angle match trial was 16 s. One (8 or 10 or 18 s) interval followed each trial, and no stimuli were presented during the interval.

### Data acquisition

Functional magnetic resonance imaging were acquired on a 3-T Siemens Trio whole-body MRI system. Standard sequence parameters were used to obtain the functional images as follows: gradient-echo EPI; repetition time (TR) = 2000 ms; echo time (TE) = 30 ms; flip angle = 85°; 32 axial slices of 3 mm thickness with 20% slice gap; Matrix = 64 mm × 64 mm; and in-plane resolution = 3.0 mm × 3.0 mm. A T1-weighted high-resolution anatomical image volume was obtained from each participant (voxel size = 1 × 1 × 1 mm^3^) before the acquisition of the functional data.

### Data analysis

Image processing and statistical analyses were performed using the Statistical Parametric Mapping package (SPM8; Wellcome Department of Cognitive Neurology, London, UK). Functional images from each run were realigned to the first data scan to correct for motion. All functional images and the T1-weighted anatomical images were then co-registered to the first scan of the tactile angle matching task. Each co-registered T1-weighted anatomical image was normalized to a standard T1 template image defined by the Montreal Neurological Institute (MNI) space. The parameters from this normalization process were then applied to each functional image. The normalized functional images were spatially filtered using a Gaussian kernel of 8 mm full-width at half maximum.

The statistical analyses of the fMRI data were conducted on two levels using the general linear model framework. The task-related neural activities under each of the three conditions were modeled with a boxcar function convoluted with a canonical hemodynamic response function. To reveal activation maps of regions specifically involved in the tactile angle matching component during Match and Non-Match tasks, we compared the two tasks with the Rest: Match—Rest and Non-Match—Rest (Figure 3). In addition, we directly contrasted the brain activity of Match and Non-Match tasks: Match—Non-Match and Non-Match—Match. Reported clusters survived an uncorrected *p* < 0.001 (height threshold *T* = 3.85) at the voxel-level and a family wise error (FWE) correction of *p* < 0.05 at the cluster-level (Friston et al., 1994; Woo et al., 2014).

**Figure 2:**
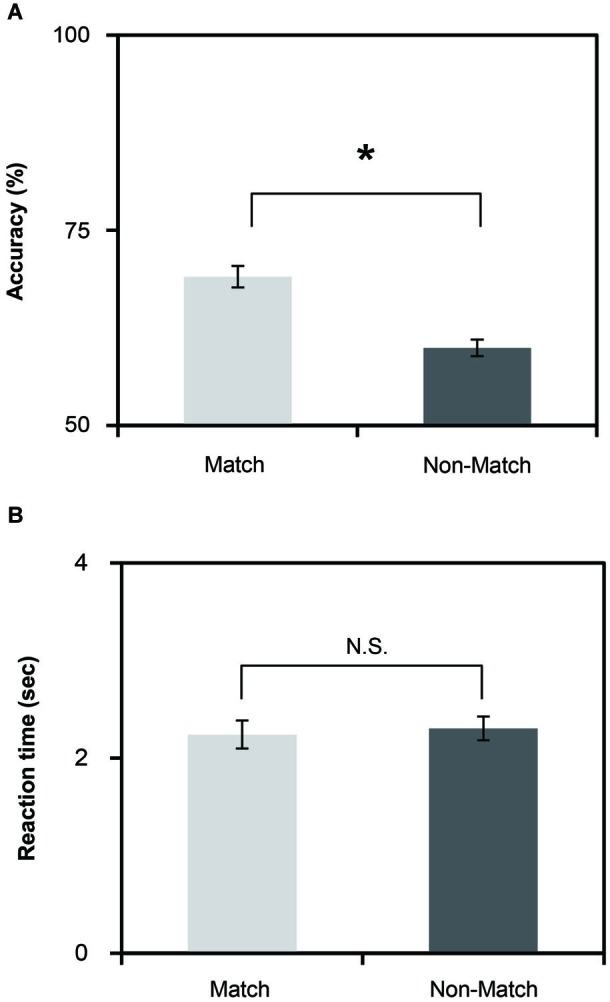
**(A)** Mean accuracies of the Match and Non-Match tasks. **(B)** Mean reaction time of the Match and Non-Match tasks. The vertical error bars represent the standard error of the mean. * represents the statistically significant of *P* < 0.05; N.S., Not Significant.

**Figure 3:**
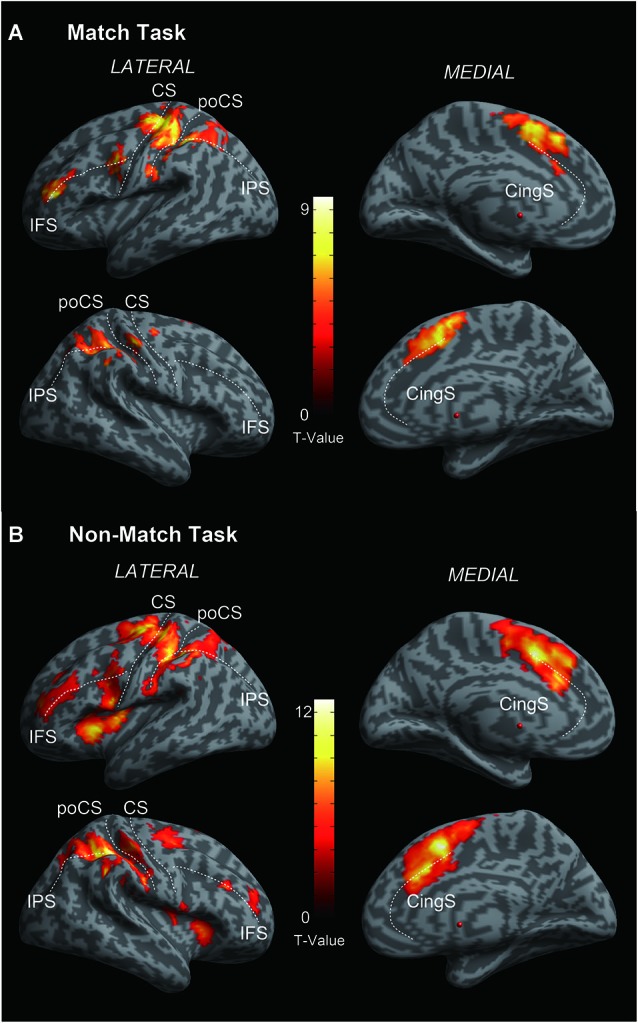
**Cortical activation of (A) Match vs. Rest and (B) Non-Match vs. Rest**. Statistical parametric maps are overlaid on a cortical surface rendering where dark gray color indicates sulcal and light gray color indicates gyral areas. Main sulci are marked with white dashed lines. IFS, inferior frontal sulcus; CS, central sulcus; poCS, postcentral sulcus; IPS, intraparietal sulcus; CingS, cingulated sulcus. FWE correction of *p* < 0.05 at the cluster-level.

## Results

### Behavioral results

To investigate the differences in task performance, we calculated the mean reaction time and accuracy for each task (Figures 2A,B). The results indicate that the Match and Non-Match tasks were challenging. The mean accuracies of all tasks exceeded the chance level. We performed paired two sample *t*-tests on the mean reaction time and accuracy. We found significant differences in accuracy times between the two tasks (*t*_(26)_ = 2.19, *P* = 0.037). The accuracy was higher for the Match task compared to the Non-Match task. However, there were no significant differences in the reaction times between the two tasks (*t*_(26)_ = −0.461, *P* = 0.648).

### fMRI results

As shown in Figures 3A,B, both of the Match and Non-Match tasks activated the bilateral postcentral gyrus (poCG), the precentral gyrus (preCG), the mFG, the PPC, the supramarginal gyrus, the cingulate gyrus, the left superior frontal gyrus (SFG), the left middle frontal gyrus (MFG), the left inferior frontal gyrus (IFG), the left angular gyrus and the right precuneus. Furthermore, the Non-Match task also activated the right IFG, MFG, SFG and the left precuneus and the bilateral parietal operculum. Moreover, we also observed activation in the bilateral parietal operculum (*x* = 54, *y* = 10, *z* = 0; *Z*-value = 4.07 and *x* = −56, *y* = 6, *z* = 4; *Z-value* = 3.69) for the Match task; however, the size of these clusters did not exceed the cluster threshold (FWE correction of *p* < 0.05). The MNI coordinates of the activated clusters (FWE correction of *p* < 0.05 at the cluster-level) and their significant *Z*-values are listed in Table 1.

**Table 1:** **Summary of the anatomical region, hemisphere, MNI coordinates (*x*, *y*, *z*) and maximal *Z*-value of significant activations**.

Anatomical region	Hemisphere	Non-Match vs. rest				Match vs. rest			
		*x*	*y*	*z*	*Z*-Value	*x*	*y*	*z*	*Z*-Value
Precentral gyrus	L	−34	−18	58	5.50	−50	4	34	4.57
	R	30	−8	52	4.14	42	−12	62	3.88
Postcentral gyrus	L	−40	−30	58	4.93	−42	−26	62	4.98
	R	38	−38	50	5.29	48	−36	50	4.93
Medial frontal gyrus	L	−12	−10	52	4.88	−6	10	56	4.79
	R	10	10	48	5.83	6	6	56	4.77
Superior frontal gyrus	L	−32	48	18	4.56	−34	52	20	4.19
	R	30	42	20	4.01
Middle frontal gyrus	L	−24	−14	58	5.08	−36	40	12	5.20
	R	30	42	22	4.19
Inferior frontal gyrus	L	−30	20	−2	4.56	−38	38	12	5.06
	R	32	26	2	3.98
Inferior parietal lobule	L	−36	−30	38	5.16	−42	−40	44	5.28
	R	40	−38	46	5.69	46	−36	46	5.03
Parietal operculum	L	−42	2	12	5.80
	R	48	0	4	4.13
Supramarginal gyrus	L	−44	−40	42	5.35	−38	−36	40	4.99
	R	44	−40	34	5.30	34	−54	36	3.98
Angular gyrus	L	−32	−56	42	4.99	−30	−54	40	5.12
Precuneus	L	−22	−66	52	3.66
	R	28	−56	54	4.65	32	−60	40	4.05
Cingulate gyrus	L	−6	8	42	5.80	−2	8	44	4.71
	R	12	26	26	5.34	6	8	44	4.80

*Note: L, left hemisphere; R, right hemisphere; *P*_(FWE)_ < 0.05 at the cluster level corrected for multiple comparisons*.

**Figure 4:**
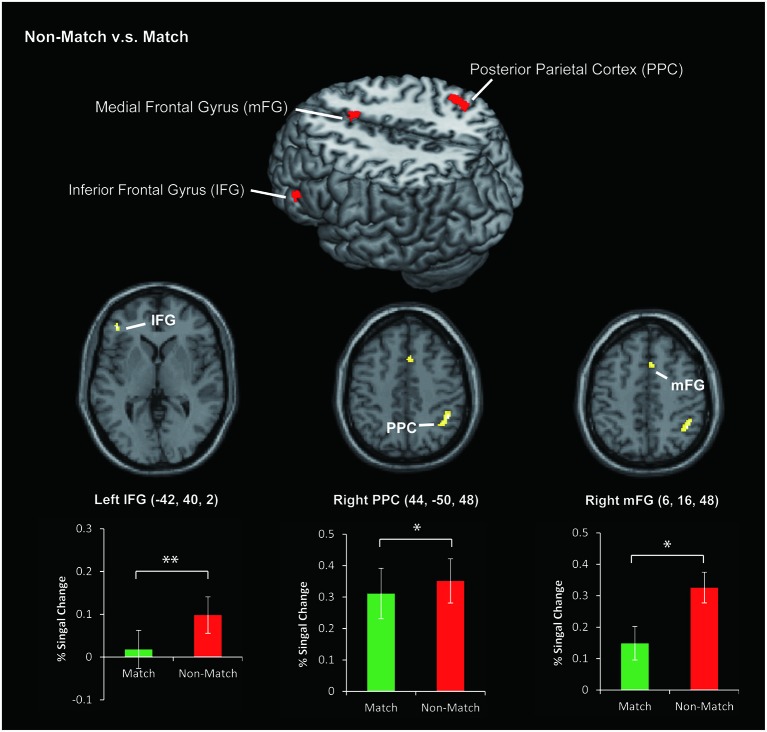
**The area of activation for Non-Match vs. Match**. The colored bar graphs indicate the task-related activation (% Signal Change) of Non-Match vs. Rest and Match vs. Rest contrasts using a volume of interest with a sphere of 8 mm diameter. The centers of the spheres were the peak coordinates of activation. The error bars indicate the standard error of the mean. *P < 0.05, **P < 0.01.

In this study, we focused on the different neural substrates of the tactile angle matching and non-matching process. To ensure the neuronal activations were not influenced by the difficulty of the task, the each subject’s accuracy was entered as a covariate in the second-level analysis. Then, we used direct Match vs. Non-Match and Non-Match vs. Match contrasts. Because we had an *a priori* hypothesis that the Non-Match task would activate the IFG, PPC and mFG regions more than the Match task, we limited our search to each of these regions, as defined by the SPM Anatomy toolbox. The statistical threshold for the spatial extent test on the clusters was set at *p* < 0.05 and corrected for multiple comparisons within the search volume. The search volume was 296 mm^3^ for the IFG (local maxima [−42, 40, 2], *P*_(SVC–FEW)_ = 0.039), 928 mm^3^ for the PPC (local maxima [44, −50, 48], *P*_(SVC–FEW)_ = 0.005) and 208 mm^3^ for the mFG (local maxima [6, 16, 48], *P*_(SVC–FEW)_ = 0.049) region. The percent of signal change was defined as the mean percentage of the BOLD signal change in each task divided by the rest periods. We plotted the histograms using the percent of signal change between stimulation and rest periods in Figure 4.

## Discussion

In this study, we used an event-related fMRI experiment to address whether the priming effect decreases the activity in cortical regions during the tactile angle matching process. As we hypothesized, both the tactile angle match and non-match tasks activated a similarly distributed cerebral network, and we revealed decreased brain activity in the left IFG, right PPC and right mFG for the match task (See Figure 4). Therefore, we suggest that tactile priming engaged repetition suppression mechanisms (Wiggs and Martin, 1998) during the tactile angle matching tasks, and this process decreased activation in these areas.

### Behavioral performance and task design

The current fMRI experiment was designed to examine the neural correlates of the tactile angle matching process and test whether the priming decreases activity in the cortical regions involved in this processing. In both the match and non-match tasks, we used three angle stimuli for the encoding phase and one angle stimulus for the recognition phase to counterbalance the tactile stimulus factors between these two tasks. The main difference between the two tasks was whether the angle stimulus for the recognition phase appeared during the encoding phase. Because the overall task accuracy was above chance level, we can assume that the attentional demands were comparable between these two tasks. Hence, contrasting the non-match task with the match task should highlight the specific processing for tactile priming perception during the tactile angle matching task.

Robust priming effects lead to faster reaction times and accurate responses (Slocomb and Spencer, 2009). As shown in Figures 2A,B, the accuracy of the match task was higher than the non-match task, but there was no significant difference in the reaction times between the two tasks. This result is understandable in the context of the instructions for the subjects. To ensure that all subjects could sufficiently perceive the angle stimulus during the recognition phase and make a correct response, we asked all subjects to make a response during a 4 s response phase. Overall, these results indicate that the priming effect influenced the tactile angle matching performance.

### Activation of tactile angle matching processing

In the current fMRI experiment, all subjects were asked to complete two typically delayed tactile angle matching tasks. We observed that these two tactile angle matching tasks mainly activated the contralateral SI in the poCG (left hemisphere), and the activations were extended to the bilateral superior, middle and inferior parietal lobule, the bilateral secondary somatosensory area (SII) in the parietal operculum and the prefrontal cortical areas (Figures 3A,B). Activity in these areas is expected during tactile tasks, as shown in previous neuroimaging studies (e.g., Bodegård et al., 2001; Newmana et al., 2005; Reed et al., 2005; Hlushchuk and Hari, 2006; Kitada et al., 2006; Miquée et al., 2008). The SI contains a somatotopic organization of body representations and is the first cortical region to be activated and involved in the perception of touch (Roland et al., 1998; Bodegård et al., 2001). Previous functional neuroimaging studies have found that SII can be activated in response to tactile stimulation (Eickhoff et al., 2007, 2008) and tactile attention (Burton and Sinclair, 2000). The activation of the bilateral prefrontal and parietal cortical areas is consistent with previous studies that demonstrated that the fronto-parietal circuit is involved in tactile WM and decision making (Stoeckel et al., 2003; Reed et al., 2005; Kostopoulos et al., 2007; Nelson et al., 2009).

### Decreased activation specific to the match task

Previous studies (Wiggs and Martin, 1998; Schacter et al., 2007) indicated that the stimulus-related decreases in activation during priming are related to the phenomenon of repetition suppression in single-cell recordings, where decreased neural responses are observed as a function of stimulus repetition. Since the early 1990s, a number of neuroimaging studies (Vuilleumier et al., 2002; Henson, 2003; Dobbins et al., 2004) have demonstrated that reductions in brain activity can be observed using fMRI. In the current study, we observed decreased activation in the left IFG, the right PPC and the right mFG for the match task compared to the non-match task.

The IFG is specifically involved in the information processing of memory and retrieves the required information (Miquée et al., 2008; Zimmer, 2008; Zanto et al., 2011). Recent neuroimaging studies (Kaas et al., 2007, 2013; Kostopoulos et al., 2007; Fiehler et al., 2008) related to haptic or tactile spatial WM also indicated that the IFG plays a key role in information retrieval, and the IFG increases its functional interaction with the posterior somatosensory areas in the parietal operculum and the PPC (Kostopoulos et al., 2007). In the present task, the subjects were required to compare the angle stimulus presented during the recognition phase with the angle stimuli presented during the encoding phase (held in short-term memory) and decide whether the angle stimulus was presented during the encoding phase. Therefore, the memory retrieval processes tested here were expected to lead to activation of the left IFG. In addition, previous studies (for review, see Schacter et al., 2007) indicated that the IFG responds invariantly to the perceptual features of stimuli and showed significant response reduction effects in both visual and auditory priming studies. Moreover, a recent study (Burton et al., 2012) using repeated vibrotactile stimuli also found decreased activation in the left IFG. Therefore, the reduced activation of the left IFG obtained in our study is consistent with the previous findings and expands on them with tactile spatial stimuli.

The PPC is located at the junction of multiple sensory regions and projects to several cortical and subcortical areas, and it plays an important role in producing planned movements (Stricanne et al., 1996; Batista et al., 1999) and tactile spatial information computations (Bodegård et al., 2001; Yang et al., 2012). Recently, the involvement of the PPC in other cognitive functions such as WM and learning have become evident. Previous studies of anatomic connections revealed that the PPC receives inputs from primary and secondary sensory modalities and has extensive reciprocal connections with the frontal and prefrontal regions, such as the IFG and the mFG. This fronto-parietal circuit is activated during sequential tactile discriminations on the basis of its role in WM (Stoeckel et al., 2003). Applying these concepts to our data, we argue the following points. In the current study, bilateral PPC activation during tactile angle matching tasks can be related to tactile spatial information computation and tactile WM. However, the tactile spatial information of the match and non-match tasks was the same. Therefore, we suggest that the difference of WM processes between the two tasks may contribute to the decreased brain activation in the right PPC. In other words, the priming effect of the match task also reduced activation in the right PPC but not in the left PPC. This interpretation of the data is supported by a previous report (Stoeckel et al., 2004) that indicated that the right PPC is predominant for tactile object discrimination and information maintenance. In contrast, left PPC activation was only seen during the delay period for tactile information maintenance. In the current study, the encoding and delay phase of the match and non-match tasks were the same. Therefore, the priming effect of the cognitive phase likely reduced the activation of the right PPC. Consistent with the previous study (Stoeckel et al., 2004), we suggest that the activation of the left PPC was used only for tactile angle information maintenance but not for discrimination and decision making processes.

Moreover, we observed a significant reduction of activation in the mFG. This area is known as a pre-supplementary motor area (pre-SMA) and occupies the medial portion of Brodmann cortical area 6. The pre-SMA is involved in various other functions, including motor, sensory and WM (for review, see Criaud and Boulinguez, 2013). The frontal cortex, including the pre-SMA and IFG, was activated with the parietal cortex during the tactile spatial WM processes. Therefore, activation of the right mFG contributes to the WM processing of tactile angle matching tasks, and the priming effect of the match task reduces activation in this area.

## Conflict of interest statement

The authors declare that the research was conducted in the absence of any commercial or financial relationships that could be construed as a potential conflict of interest.
